# An explainable framework for the relationship between dementia and metabolism patterns

**Published:** 2026-01-28

**Authors:** C. Vázquez-García, F. J. Martínez-Murcia, F. Segovia, A. Forte, J. Ramírez, I. Illán, A. Hernández-Segura, C. Jiménez-Mesa, J. M. Górriz

**Affiliations:** a Department of Signal Processing and Biomedical Applications, University of Granada, Granada 18071, Spain; b Department of Statistics and Operations Research, University of Valencia, Valencia 46010, Spain; c Department of Communication Engineering, University of Malaga 29071, Spain

**Keywords:** Alzheimer, Computational Neuroscience, PET, Variational Autoencoder, ADNI

## Abstract

High-dimensional neuroimaging data poses a challenge for the clinical assessment of neurodegenerative diseases, as it involves complex non-linear relationships that are difficult to disentangle using traditional methods. Variational Autoencoders (VAEs) provide a powerful framework for encoding neuroimaging scans into lower-dimensional latent spaces that capture meaningful disease-related features. In this work, we propose a semi-supervised VAE framework that incorporates a flexible similarity regularization term designed to align selected latent variables with clinical or biomarker measures related to dementia progression. This approach allows adapting the similarity metric and the supervised variables according to specific goals or available data. We demonstrate the framework using Positron Emission Tomography (PET) scans from the Alzheimer’s Disease Neuroimaging Initiative (ADNI) database, guiding the model to capture neurodegenerative patterns associated with Alzheimer’s Disease (AD) by maximizing the similarity between the first latent dimension and a clinical cognitive score. Leveraging the supervised latent variable, we generate average reconstructions corresponding to different levels of cognitive impairment. A voxel-wise General Linear Model (GLM) reveals reduced metabolism in key brain regions, predominantly in the hippocampus, and within major Resting State Network (RSN)s, particularly the Default Mode Network (DMN) and the Central Executive Network (CEN). Further examination of the remaining latent variables show that they encode affine transformations—rotation, translation, and scaling—as well as intensity variations, capturing common confounding factors such as inter-subject variability and site-related noise. Our findings indicate that the framework effectively extracts disease related patterns aligned with established Alzheimer’s biomarkers, providing an interpretable and adaptable tool for studying neurodegenerative progression.

## 1. Introduction

Neurodegenerative Diseases (NDDs), such as AD, are characterized by progressive brain atrophy and cognitive decline. The global increase in life expectancy is contributing to a rising prevalence of NDDs, with AD and other dementias showing a 168.7% increase between 1990 and 2021 Steinmetz et al. (2024). These diseases profoundly affect individuals’ autonomy and quality of life, while also placing significant emotional, social, and financial burdens on caregivers and healthcare systems. Consequently, there is an urgent need for effective tools that enable prognosis and treatment.

Neuroimaging provides valuable insights into the neural alterations underlying these disorders; however, extracting meaningful patterns from its high-dimensional structural and functional data remains a complex task typically requiring expert interpretation. This limitation hampers early intervention, as AD is often diagnosed only when the damage is already substantial and treatment options are limited Jack et al. (2010). By identifying subtle alterations in brain structure and function, imaging techniques can help characterize disease patterns, track progression trajectories, and potentially distinguish high-risk individuals at preclinical stages Frisoni et al. (2010).

Given these challenges, dimensionality reduction techniques have become essential for the analysis of neuroimaging data. This high-dimensionality makes it difficult to identify disease-related patterns, as relevant information is often embedded within complex, non-linear relationships. The *manifold hypothesis* Tenenbaum et al. (2000); Cunningham and Yu (2014); Bengio et al. (2013) suggests that, despite the apparent high-dimensionality of the neuroimaging data, meaningful representations lie on a lower dimensional manifold. By mapping neuroimaging data to a structured latent space, we can uncover disease-related patterns that might otherwise remain hidden in raw imaging data.

In this context, numerous studies have explored both linear and non-linear approaches to uncover latent representations in neuroimaging data, aiming to better characterize neurodegenerative processes. Early works based on linear generative models, such as Dukart et al. (2013), used GLM-based frameworks to describe atrophy and hypometabolism in Fluorodeoxyglucose Positron Emission Tomography (FDG-PET) and Structural Magnetic Resonance Imaging (sMRI) data, providing valuable insights despite inherent limitations in modeling complex non-linear patterns.

To address the complex and non-linear data, we use VAEs, a type of generative model that offers significant advantages Kingma et al. (2013). VAEs allow us to learn representations of data, which we can map back into the brain space. This capability enables the analysis of how specific latent patterns correspond to variations in brain structures, making it a powerful tool for studying neurodegeneration patterns. Additionally, VAEs are particularly suited for this task due to their explicit nature, which allows direct access to the variables of the learned latent distribution Dalca et al. (2019), facilitating the analysis of potential relationships between latent representations and clinical biomarkers.

The standard VAE framework assumes a generative model p(x,z) that captures the joint distribution between the observed data X and a hidden space Z. The VAE learns to map from X onto a latent manifold, where smooth transitions along the manifold correspond to smooth changes in the learned latent variables, which encode subject-specific characteristics. The encoder learns an approximate posterior that maps each observation to a distribution over latent variables. As a result, the model organizes the latent space such that similar data points are placed nearby, and traversing specific directions in the manifold leads to interpretable smooth variations in the latent features.

Several works have explored the application of VAEs to neuroimaging data for dementia research. Wakefield et al. (2024) applied a graph-based unsupervised VAE to FDG-PET scans, achieving high accuracy in distinguishing AD patients from controls while enhancing explainability through latent representations. Similarly, Dolci et al. (2024) built a classification model using both genetic and neuroimaging data, extracting features through a VAE. Their model achieved high accuracy and unraveled key regions for the classification task. In a related approach, Kumara et al. proposed a multimodal VAE for normative modeling of brain MRI, successfully capturing deviations associated with disease progression.

Despite these advances, unsupervised VAEs often struggle to disentangle clinically meaningful features from noise, limiting their interpretability—there is no guarantee as to what each latent variable encodes Locatello et al. (2019); Eastwood and Williams (2018). This has motivated the study of semi-supervised variants, where guidance from clinical variables helps shape more interpretable latent spaces. Zhao et al. (2019) proposed a supervised VAE that integrates an auxiliary regressor to guide the latent space towards predicting age from MRI scans, enabling a more meaningful and structured disentangled latent representation. Moreover, in our prior work on Parkinson’s Disease Vázquez-García et al. (2024), we demonstrated the ability of VAEs to extract clinically relevant information from FDG-PET scans. Latent representations were shown to encode symptomatology, providing evidence that generative models can capture disease-relevant patterns from neuroimaging data.

However, these works do not directly disentangle latent patterns of dementia symptomatology, which is essential for understanding disease heterogeneity and progression. In this work, we propose a semi-supervised VAE framework, guiding the latent space towards capturing dementia-related patterns, by introducing a similarity regularization term between the latent space and dementia symptomatology. The main contributions of this work are as follows. First, a semi-supervised VAE framework is proposed, in which latent dimensions are explicitly guided to capture dementia-related symptom patterns through a similarity regularization term. Second, we provide an analysis of the convergence behavior of the model, identifying conditions that prevent both mean collapse and the collapse of regularization terms. Third, we leverage the generative capabilities of the model to map neurodegenerative patterns into brain space, yielding voxel-wise representations that affect how dementia information is encoded in the latent space. Finally, we design an interpretable mechanism to represent common confounding factors—such as intersubject variability and acquisition noise—within the model.

## 2. Materials and Methods

### 2.1. Data description

Data used in the preparation of this article were obtained from the ADNI database (adni.loni.usc.edu). The ADNI was launched in 2003 as a public-private partnership, led by Principal Investigator Michael W. Weiner, MD. The original goal of ADNI was to test whether serial MRI, PET, other biological markers, and clinical and neuropsychological assessment can be combined to measure the progression of mild cognitive impairment (MCI) and early AD. The current goals include validating biomarkers for clinical trials, improving the generalizability of ADNI data by increasing diversity in the participant cohort, and to provide data concerning the diagnosis and progression of AD to the scientific community. For up-to-date information, see adni.loni.usc.edu.

We selected a subset containing 3466 FDG-PET scans, which measure brain glucose metabolism, a key indicator of neurodegeneration Mosconi (2005). In addition to imaging data, we included structural biomarker volumes (Hippocampus, Medial temporal lobes, Entorhinal cortex, and Fusiform), normalized by brain size to reduce inter-subject variability.

PET scans were coregistered to a common template using rigid-body transformation (SPM12 Ashburner et al. (2014)), but not spatially normalized, in order to preserve individual anatomical variability for the convolutional architecture. Intensity normalization was conducted through a two-step process: min-max normalization (relative to the 99th percentile), followed by exponential transformations to enhance contrast. We found this normalization pipeline to be the best performing and informative about relevant brain structures, specially in the cortex.

### 2.2. Model description

A VAE consists of two components: an encoder network, which approximates the posterior distribution q(z∣x), mapping the input data x into a latent representation z; and a decoder network, which reconstructs the data through the likelihood model p(x∣z).

Training is performed by maximizing the marginal log-likelihood log p(x) of the data, which is generally intractable. Instead, the model optimizes a variational lower bound Kingma et al. (2013), known as the Evidence Lower Bound (ELBO). The ELBO can be written as:

(1) $$𝓛(x)=\underset{{𝓛}_{\text{recon}}}{\underset{︸}{E_{z∼q(z|x)}[p(x|z)]}}-\underset{{𝓛}_{\text{KL}}}{\underset{︸}{D_{KL}(q(z|x)\|p(z))}}={𝓛}_{\text{recon}}-{\text{L}}_{\text{KL}},$$

where the first term ${𝓛}_{recon}$ corresponds to the reconstruction accuracy, i.e., how well the decoder can reconstruct the input data from the latent representation, and the second term is the Kullback-Leibler (KL) divergence between the approximate posterior and the prior p(z), which regularizes the latent space to follow the prior distribution.

For the purpose of the experiments, we opted to use the Mean Square Error (MSE) as the regularization loss, given its suitability for reconstructing continuous-valued neuroimaging data:

(2) $${𝓛}_{MSE}=\frac{1}{N}\sum_{i=1}^{N}{\left\|x_{i}-{\hat{x}}_{i}\right\|}^{2}.$$

However, a different choice of reconstruction error—Structural Similarity Index (SSIM), Binary Cross-Entropy (BCE), etc.—is also valid, as long as it is appropriate for the type of data handled. On the other hand, we chose a specific expression for the KL divergence, by choosing gaussian formulas for both prior p(z) and posterior q(z∣x):

(3) $$D_{KL}(q(z|x)\|p(z))=\frac{1}{2}\sum_{i=1}^{d}\left(1+\log\left(\sigma_{i}^{2}\right)-\mu_{i}^{2}-\sigma_{i}^{2}\right),$$

where d is the latent space dimensionality, and $\mu_{i}$ and $\sigma_{i}^{2}$ are the mean and variance of the i-th variable. Additionally, we employ the Beta-VAE Higgins et al. (2017), which introduces an extension to the original ELBO by incorporating a weighting parameter β to the divergence loss, such that the regularization of the latent space, $\beta {𝓛}_{\text{KL}}$, is now controlled by this parameter. Note that the choice of the KL divergence is not unique, and that other divergences can be used—the Maximum Mean Discrepancy (MMD), for instance Zhao et al. (2017).

In our framework, in addition to the traditional ELBO objective, we also incorporated an additional third loss that encourages that a set—one or more variables—of the latent space variables capture relevant patterns of cognitive impairment. We denote this term as ${𝓛}_{\text{similarity}}$, which measures the statistical similarity between the set of latent variables and the external variable (dementia score, region volume, etc). The choice of metric to quantify similarity is flexible and can be adapted for the case: Pearson correlation, mutual information, or other functions are valid options. Likewise, the external variable used to compute the similarity can be chosen to be different types of biomarkers, clinical scores or other relevant metrics. Formally, we define this regularization term as:

(4) $${𝓛}_{\text{similarity}}=𝒟\left(z_{\left(k\right)},y\right),$$

where $z_{\left(k\right)}$ is the set of supervised variables, y is the external variable of interest, and 𝒟(⋅,⋅) is a general similarity metric. In our particular experimentation, we considered the Pearson correlation between the first latent variable $z_{0}$ and the cognitive score Alzheimer’s Disease Assesment Scale (ADAS13), which results in the following expression for the similarity loss:

(5) $${𝓛}_{\text{similarity}}=-\frac{\sum_{i=1}^{N}\left(z_{0}^{i}-{\bar{z}}_{0}\right)\left(y_{i}-\bar{y}\right)}{\sqrt{\sum_{i=1}^{N}{\left(z_{0}^{i}-{\bar{z}}_{0}\right)}^{2}}\sqrt{\sum_{i=1}^{N}{\left(y_{i}-\bar{y}\right)}^{2}}},$$

where N is the number of subjects, $z_{0}^{i}$ denotes the first latent variable of the i-th subject, $y_{i}$ is the value of the clinical score for the i-th subject, and $\bar{z}$ and $\bar{y}$ are the mean values of the first value of the latent space, and the clinical score, respectively.

The final total loss function used during training was:

(6) $$𝓛={𝓛}_{MSE}-\beta {𝓛}_{KL}+\alpha {𝓛}_{similarity},$$

where we added α as hyperparameter to control the strength of the similarity regularization.

### 2.3. Architecture

The architecture of the semi-supervised 3D convolutional VAE is as follows:

- **Encoder:**
  - Four convolutional layers with kernel sizes of 11, 7, 5, and 3 (with 32, 64, 128, and 256 channels, respectively), progressively decreasing in size, each followed by a ReLU activation function and batch normalization to ensure stable training and improve convergence.
  - A fully connected network transforms the output of the last convolutional layer, which has 9216 neurons (256 channels ×3 × 4 × 3 spatial dimensions), into a linear feature representation of 256 neurons, followed by ReLU activation.
  - Two separate fully connected layers map this representation into a mean space and a log-variance space. These are then reparameterized to obtain the final latent space representation.
- Decoder:
  - A fully connected network maps the latent space back into a linear feature representation of 4608 neurons (128 channels ×3 × 4 × 3 spatial dimensions), followed by a ReLU activation.
  - The decoder employs three transposed convolutional layers with increasing kernel sizes of 3, 4, and 11 (with 128, 64, and 32 channels, respectively), each followed by a ReLU activation function and batch normalization to progressively reconstruct the input volume.

The model was trained using the PyTorch library, with Adam as the optimizer and a learning rate of 2 × 10^−5^. The hyperparameters, including the number of epochs, β,α, batch size, and latent space dimensionality were selected based on preliminary experiments. The dataset was split into training, evaluation, and testing sets with respective proportions of 0.65, 0.15, and 0.2.

### 2.4. Model Evaluation and Interpretability

We explored the hyperparameter space to identify the most suitable value ranges for the regularization parameters β and α, as well as the dimensionality of the latent space. To support this analysis, we constructed a set of phase diagrams that illustrate the model’s behavior under varying hyperparameter configurations. Phase diagrams—commonly used in physics and engineering—serve here to visualize how changes in these parameters affect model performance, by showing how the behavior changes.

In our context, these phase diagrams reveal distinct operational regimes of the VAE, capturing regions where the model successfully learns meaningful latent representations—which we denote as the *stable regime*—versus regions where the model collapses to trivial solutions—denoted by the *failure regime* (e.g., posterior collapse to the mean of the input data). Our phase diagrams clearly delineate the hyperparameter regions where this collapse happens versus those where the model learns useful, informative latent representations. This insight is crucial for tuning VAEs in neuroimaging, where avoiding posterior collapse ensures the latent space captures meaningful biological variability rather than trivial averages.

To quantify this we compute a first phase diagram, where we analyze the distribution of the mean latent vectors across the validation set. Specifically, we compute the average Euclidean distance of each latent mean vector to the global centroid of all latent means. This metric captures dispersion of the representations in the latent space: higher average distances indicate more diverse and informative encodings, whereas low values suggest that the latent variables have collapsed towards a single point, reflecting a non-informative utilization of the latent space. By studying how this dispersion varies with the latent space dimensionality and the strength of the KL regularization, we identify the stable regimes versus the failure regimes. The computed Euclidean distance metric is given by the formula:

(7) $${𝒟}_{\mu }=\frac{1}{N}\sum_{i=1}^{N}{\left\|\mu_{i}-\bar{\mu }\right\|}_{2},$$

where $\bar{\mu }=\frac{1}{N}\sum_{i=1}^{N}\mu_{i}$ is the centroid of all the latent mean vectors $\mu_{i},\|\cdot {\|}_{2}$ is the euclidean distance, and N is the number of subjects.

Next, the second phase diagram shows whether the semi-supervised model learns latent representations that are informative about dementia, by varying the hyperparameters β and α. This analysis explores how the interplay between the KL divergence and the similarity regularization terms affects the model’s behavior. Specifically, we compute the Pearson correlation between the first latent dimension $z_{0}$ and the cognitive score across different β and α values to identify the stable regimes—where model captures meaningful associations with dementia severity—and failure regimes —where the similarity is either negligible or overly dominant, leading the model to converge to a non-informative minimum. While we use Pearson correlation in this case, the approach can be generalized to any similarity metric defined in equation (4).

Notice that, while we use the same terminology (stable/failure regimes) for consistency, we emphasize that these regimes reflect different failure modes: either a loss of latent variability due to posterior collapse, or a loss of clinical relevance due to the ineffective or overly strong similarity regularization. Importantly, both conditions must be satisfied for the framework to be considered effective.

To further characterize the learned latent space, we systematically sample from it to generate synthetic subjects and examine the resulting reconstructions. Specifically, we generate latent representations of the form $\left(z_{0},𝒩(0,1)\right)$, simulating subjects who share the same level of dementia severity $\left(z_{0}\right)$ but differ due to inter-subject variability. These codes are mapped back to brain space and averaged to reconstruct mean PET scans, which reflect characteristic brain patterns associated with each level of dementia. To analyze how these generated brain patterns evolve with dementia severity, we apply a voxel-wise GLM Nelder and Wedderburn (1972) to the reconstructed scans. This allows to capture intensity variations as a function of dementia severity and produce statistical maps that highlight visual neurodegeneration progression patterns.

Additionally, to quantitatively assess the relationship between the model and dementia diagnosis, we conduct a binary logistic regression to distinguish between AD subjects and Healthy Controls (HC). To ensure the robustness of the results, we employ a bootstrap validation Efron and Tibshirani (1993) approach, repeating the procedure 10 times and reporting the average perforance. Building upon this, classification was performed using three distinct input sets: (a) similarity-related variables only, (b) the remaining latent space variables, and (c) the cognitive score. We further compared their classification performances through boxplots to illustrate the differential predictive power of each input set.

Finally, to further explore and interpret the latent space, we perform a systematic variation along each individual latent dimension. Starting from a latent vector initialized to zero, we vary one latent variable at a time across its observed range and map the resulting vectors back to brain space. This enables us to visualize how changes in each specific latent variable influence the reconstructed brain images, providing a visual insight into the role of each dimension.

## 3. Results

### 3.1 . Exploration of the Hyperparameter Space

**Figure 2:**
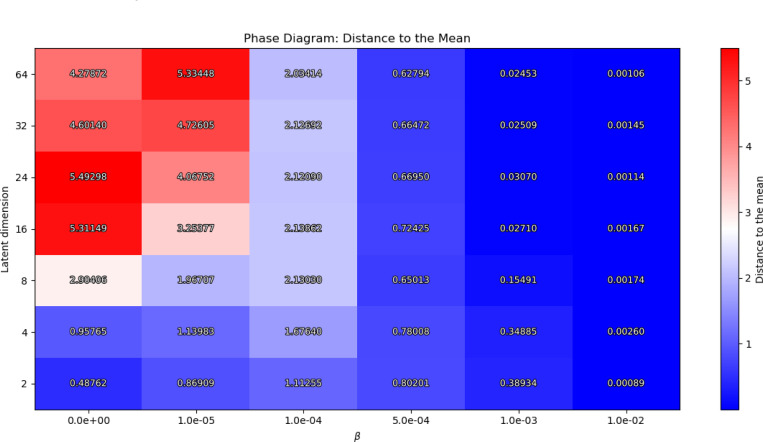
Phase diagram of latent dimensionality vs. β. The color code shows the mean euclidean distance to the mean ${𝒟}_{\mu }$ of the latent variables. Large values of β cause the model to collapse to the mean, whereas small values of latent dimension are not sufficient to capture the variability of the data.

**Figure 3:**
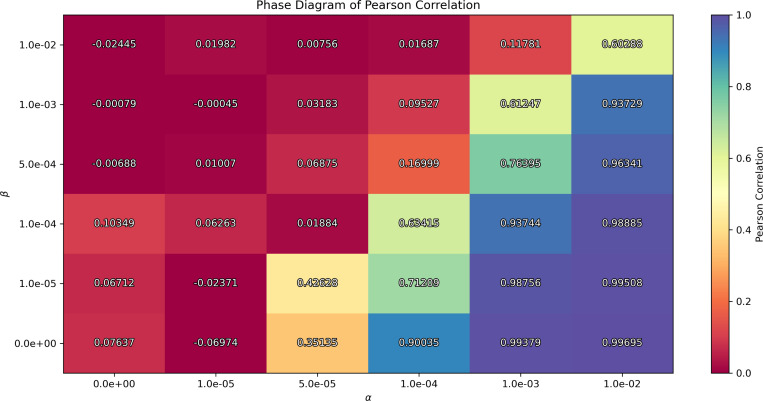
Phase diagram of KL divergence vs. Pearson regularization. The color code shows the convergence of the Pearson correlation. Large values of α lead to non-informative regularization of the latent space, whereas small values do not produce patterns correlated with dementia. The latent dimensionality is constant at 8.

To examine model convergence and parameter ranges, figures 2 and 3 present the resulting phase diagrams. In the first figure, the color scale represents the mean euclidean distance to the mean of the latent representation across dimensions, computed using equation (7). Here, we fix α=0 to isolate the effect of KL regularization on the model’s convergence behavior.

Three distinct regions emerge in this first phase diagram, depending on the ${𝒟}_{\mu }$ values. First, a deep blue region indicates very low deviation from the mean, suggesting model collapse to the data mean and therefore a failure regime. This region is associated with either high β values (strong KL regularization) or small latent dimensions. Second, a red region reflects high variance, indicating well-separated latent representations with large variability. This region indicates that the model acts almost like a deterministic autoencoder, also corresponding to a failure regime. Finally, a third intermediate region, corresponding to light colors transitioning from blue to pink, shows the stable regime, where representations exhibit variability but remain close within the latent space.

The second phase diagram displays the convergence values of the Pearson correlation between the first latent variable $z_{0}$ and the cognitive score (ADAS13), as a function of the regularization parameters β and α, with the latent dimensionality constant. We identify three distinct regions. The red region corresponds to near-zero correlation, indicating that the latent space fails to capture clinically relevant information, and thus a failure regime. In contrast, the bluish-purple region shows correlation close to one, suggesting that the similarity regularization is overly dominant, providing non-informative representations, which corresponds to a failure regime. We identify the stable regime in the intermediate transition corresponding to light values from orange to blue, where the model achieves meaningful correlations that align with clinical information.

Now, to conduct our experiments and analyze the resulting representations generated by the model, we selected an appropriate set of hyperparameters. The results presented in this work were obtained using the following set of hyperparameters: a latent dimension of 8, a learning rate of 2 × 10^−5^, a batch size of 8, $\beta =1\times 10^{-4}$, and $\alpha =1\times 10^{-4}$. It is worth noting, however, that similar values of these parameters yield comparable results, provided they lie within the stable regimes shown in Figs. 2 and 3, due to the flexibility of the model.

We encoded the input volumes of the test set, obtained their latent codes, and decoded back to brain space, to see the resulting reconstructions, shown in 4. In this figure we present input slices (odd columns) along with their corresponding reconstructions (even columns).

### 3.2 . Dementia related-Patterns

**Figure 5:**
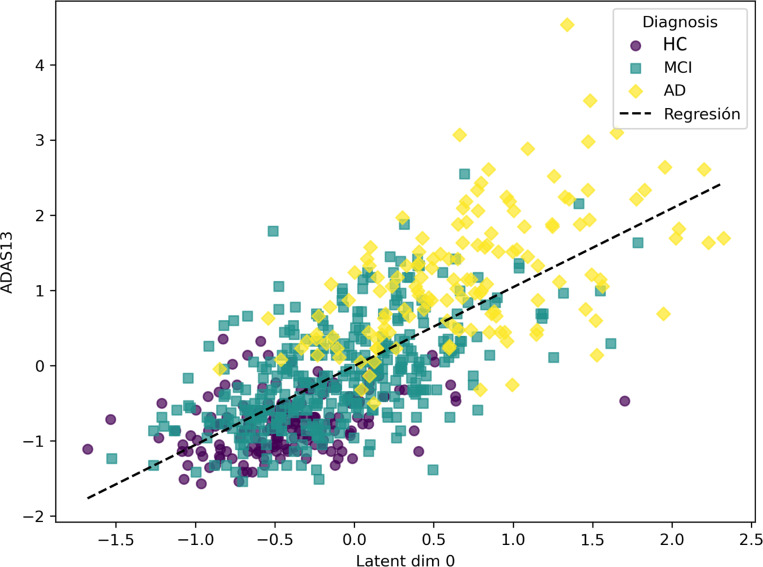
Relationship between the value of the latent variable $z_{0}$ (test set) and the ADAS13 score. r=0.790,p≪0.001.

The semi-supervised VAE effectively structured its latent space aligning ADAS13 scores, with the first latent dimension, $z_{0}$, capturing the trajectory of dementia progression. As illustrated in Fig. 5, $z_{0}$ arranged the test subjects along a continuum reflecting cognitive decline, indicating that the model successfully learned a clinically meaningful representation of disease severity. The color code shows the diagnosis of the subjects: HC, Mild Cognitive Impairment (MCI), and AD.

**Figure 6:**
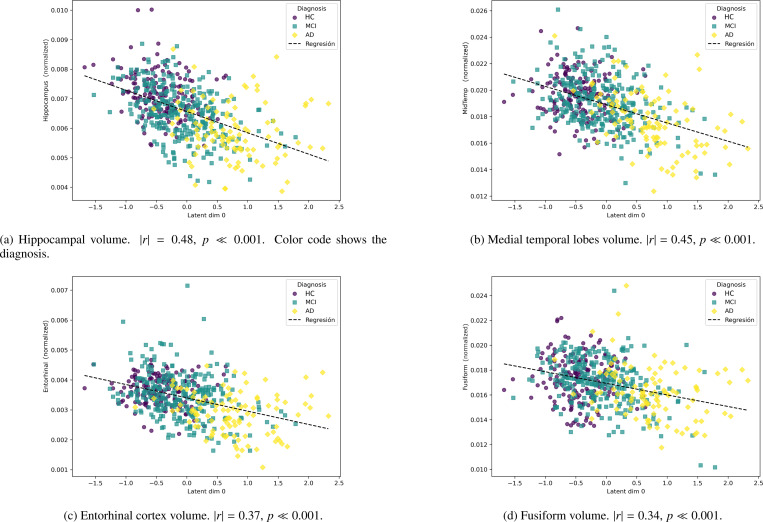
Correlation between latent representations of test set against the latent variable $z_{0}$, for several biomarker measures. All volume measures are normalized by the brain size.

Additionally, we examined the relationship between $z_{0}$ and several key biomarkers of dementia. As shown in Fig. 6, brain regions typically affected in AD—such as the hippocampus Jack Jr et al. (2008), medial temporal lobes Wuestefeld et al. (2024), entorhinal cortex Igarashi (2023), and fusiform gyrus—exhibited varying degrees of correlation with $z_{0}$. Notably, the hippocampus and medial temporal regions displayed good associations, while the fusiform gyrus showed only a weak correlation Braak and Braak (1991).

**Figure 7:**
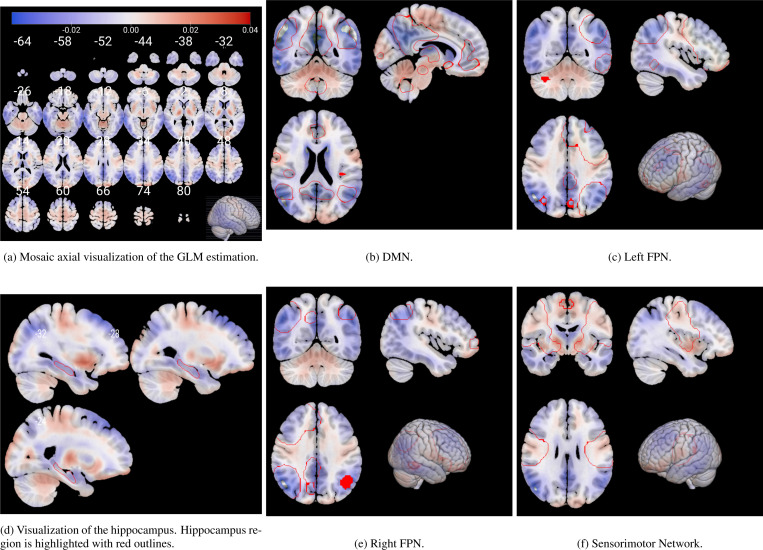
Voxel coefficients estimated by the GLM model between metabolism and disease severity. (a) shows a general visualization of the whole brain across different slices. (d) highlights in red the hippocampus ROI across several slices. (b), (c), (e) and (f) highlight some relevant RSNs. Visualization is performed using the MRIcroGLM sofware Rorden (2017).

To further investigate the biological relevance of $z_{0}$, we generated average latent representations corresponding to fixed values of this variable within its observed range (see Fig. 5). These representations were decoded into brain space, and a voxel-wise GLM was applied to identify structural changes. The resulting statistical map (Fig. 7) highlights regions most affected by neurodegeneration. Decreased metabolism (in blue) was predominantly observed in the prefrontal and medial temporal cortices, as well as in parts of the occipital lobe. In particular, Fig. 7d reveals a well-defined decline in metabolic activity in the hippocampal region. In contrast, increased metabolism (in red) was found in the motor cortex and various subcortical structures, as found in literature Mosconi (2005); Minoshima et al. (1997); Robert B. Daroff (2016).

Additional insights are provided in Figs. 7b, 7c, 7e, and 7f, which show the voxel-wise GLM coefficients overlaid on key RSNs. These include the DMN and the left and right Fronto-Parietal Networks (FPNs). Both the DMN and FPNs demonstrated significant reductions in metabolic activity, whereas the Sensorimotor Network exhibited either no significant changes or slight increases in metabolism.

### 3.3. AD classification

**Figure 8:**
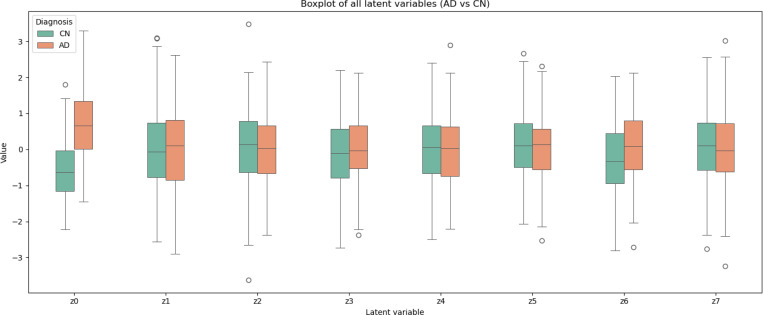
Classification performance (AD vs. HC). The figure shows the contribution of each latent variable $\left(z_{\left(k\right)},k=0,\ldots 7\right)$ to the prediction.

**Figure 9:**
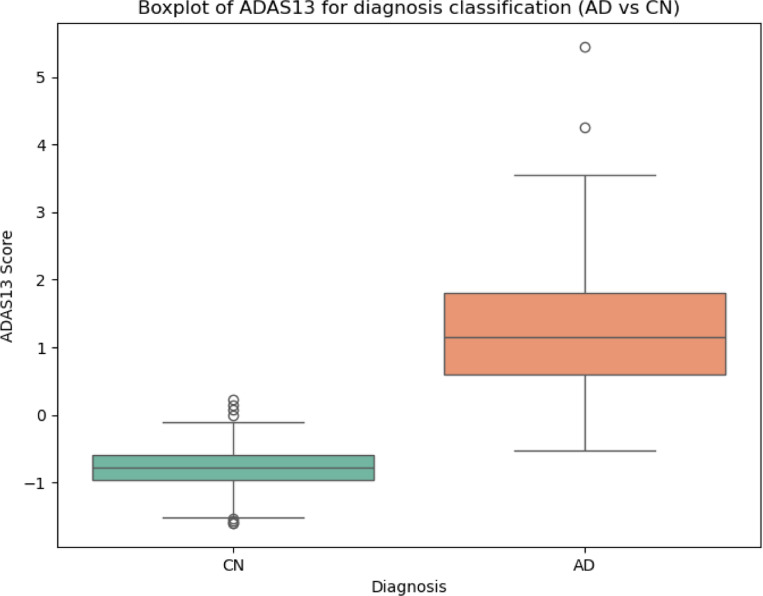
Classification based on the dementia score ADAS13.

Finally, to quantitatively assess the discriminative power of the learned representations, we performed binary logistic regression to classify AD patients and HC, using the latent representations of the test set as input. The classification results are summarized in Table 1. Additionally, in Fig. 8 we show the individual contribution of each latent variable to the classification task. Here, we visualize the contribution of each dimension separately through boxplots. We find that the similarity-related latent variable $z_{0}$ accounts for most of the discriminative power. The remaining latent variables $\left(z_{\left(k\right)},k=1,\ldots 7\right)$ show limited contribution. For comparison, we also include the classification performance obtained using the cognitive score (ADAS13) as the only input (Fig. 9). As expected, ADAS13 achieves high predictive performance, since it serves as a clinical benchmark for disease diagnosis.

### 3.4. Confounders —Exploration of the latent space

**Figure 10:**
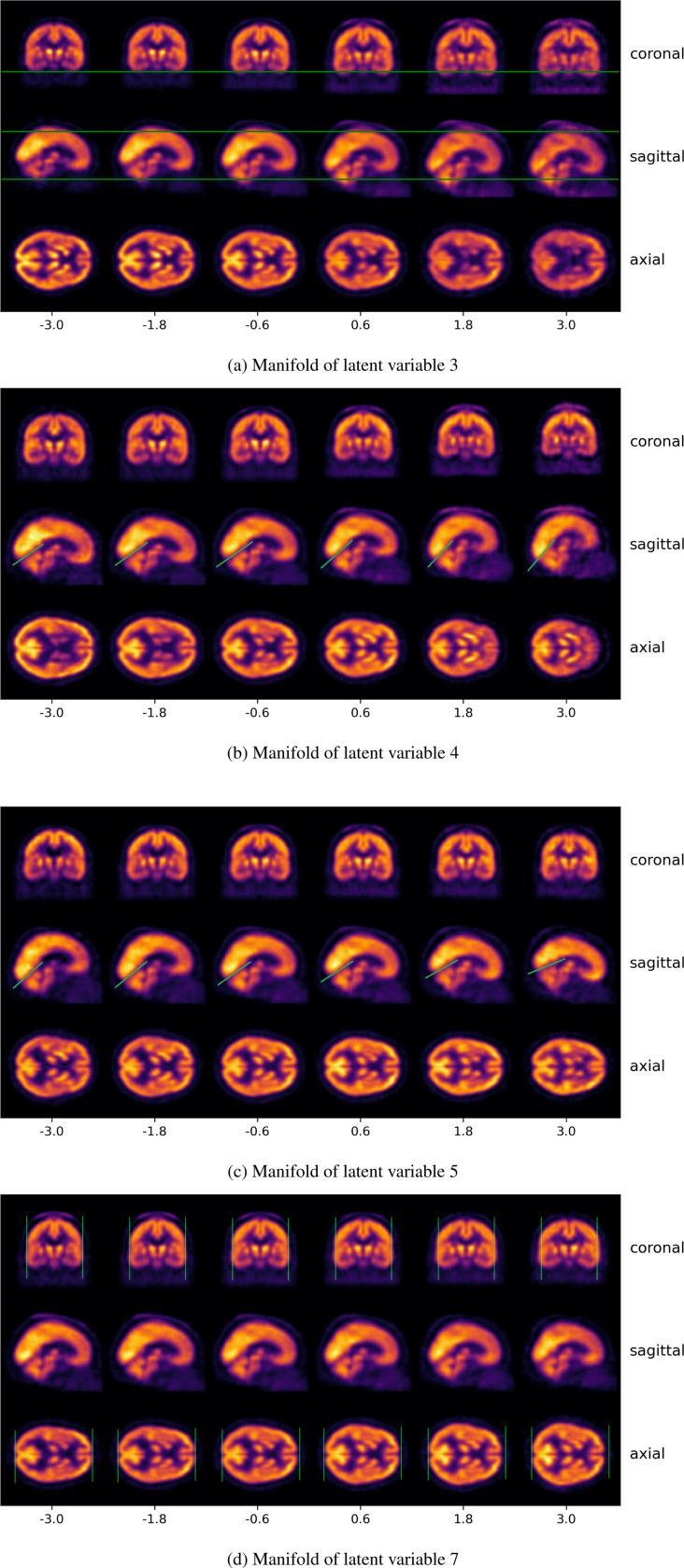
Reconstruction of the manifold for different latent variables. We observe that affine transformations are encoded within the confounder variables. (a) depicts a translation along the Z-axis, clearly visible in the sagittal plane. (b) and (c) show counterclockwise and clockwise rotations, respectively, also observable in the sagittal plane and highlighted by the green line over the tentoriym cerebelli region. (d) illustrates a scaling effect, where elongation occurs along both the X and Y axis.

In order to understand how other biological and noise factors are incorporated within this framework, we now explored the remaining variables of the latent space. In fig. 10a, we show the effect of modifying latent variable 3: positive values lead to a downward displacement along the Z-axis, whereas negative values correspond to upward shifts.

Similarly, Fig. 10c demonstrates that latent variable 5 encodes a rotation along the Y axis. This effect is particularly visible in the orientation of the tentorium cerebelli—the membrane that separates the cerebrum from the cerebellum in the occipital lobe—, which rotates clockwise as the latent value increases. An analogous but opposite transformation is observed for latent variable 4 (Fig. 10b), which induces a counterclockwise rotation in the same axis, again discernible through the position of the tentorium cerebelli.

In contrast, latent variable 7 (Fig. 10d) appears to encode a shape transformation. Negative values of this variable produce more elongated brains, whereas positive values result in shorter, more compact morphologies.

Overall, we find that most latent dimensions predominantly encode affine transformations, including translations (e.g., Fig 10a), rotations (e.g., Figs. 10b and 10c), and scaling effects (e.g., 10d). Furthermore, the colormap of the reconstructions indicates that the latent variables encode not only structural information, but also intensity-related variations. For example, positive values of latent variable 3 are associated with a more sharply defined skull and reduced contrast between the brain and surrounding tissue. In contrast, negative values enhance the contrast between the brain and the background structures, producing a skull-stripping effect. Additionally, we observe a general decrease in intensity in specific brain regions, which may reflect reduced tracer uptake.

## 4. Discussion

In this study, we explored the application of a semi-supervised VAE for analyzing AD patterns using PET scans from the ADNI database. Our primary objective was to understand how complex patterns of disease severity are encoded in the latent space and how this space organizes subjects according to their degree of dementia. To encourage the model to learn informative latent representations, we introduced a regularization strategy based on a similarity loss—(4) controlled by the hyperparameter α—that guides a set of latent variables to capture features related to dementia severity. In particular, to conduct the experiments, we chose the similarity function to be the Pearson correlation, defined by (5), between the first latent variable and the cognitive score ADAS13.

First, we conducted an exploratory study to ensure the model was effectively utilizing the latent space. The resulting figures 2 and 3 provide us with a proxy for selecting hyperparameters that avoid posterior collapse to the mean and provide informative representations of neurodegeneration progression. This analysis is crucial for the study of VAEs in neuroimaging, as complex data is sensitive to hyperparameters, as shown in the figures.

Next, we selected a fixed set of hyperparameters and studied the resulting latent representations of the model, demonstrating the model’s ability to encode dementia patterns that might otherwise go unnoticed in neuroimaging data (Fig. 5). As shown in Fig. 6, several key biomarkers associated with AD exhibit correlation with the dementia-related variable. Notably, hippocampal volume and medial temporal lobe volume demonstrate a particularly strong association. In contrast, the fusiform gyrus shows only a weak correlation, which is consistent with its involvement in later stages of the disease Braak and Braak (1991).

The generative nature of the VAE allowed us to map these dementia patterns back to brain space, providing an interpretable framework for visualizing the patterns learned by the model to distinguish between AD subjects and normal controls. By performing a GLM on reconstructions of subjects with different values of the first latent variable, we were able to visualize the variations due to dementia in the brain space (Fig. 7). We found that the patterns learned by the model correspond to well-known regions affected by the disease. A marked reduction in glucose metabolism is observed within the DMN —a well-established hallmark of AD Greicius et al. (2004); Buckner et al. (2008), as seen in Fig. 7b. Similarly, Figs. 7c and 7e show that the CEN, which encompasses both FPNs, also shows reduced metabolic activity, in line with previous literature. The DMN is involved in internally directed cognitive processes such as introspection and memory retrieval, while the CEN supports functions like attention, working memory, and cognitive control Raichle et al. (2001); Seeley et al. (2007). These domains are significantly impaired in AD Sorg et al. (2007); Zhou et al. (2010); Brier et al. (2014), making the observed metabolic patterns both biologically plausible and interpretable.

In contrast, the Sensorimotor Network —typically preserved in AD—shows either a slight increase or no significant change in metabolic activity. These patterns are clearly localized by our model mainly to the precentral and postcentral gyri, areas responsible for voluntary motor control and somatosensory processing Mosconi (2005); Sperling et al. (2010). We observe a similar behavior in the central occipital cortex, which is also known to be unaffected by AD Scahill et al. (2002).

Furthermore, we found that the remaining variables in the latent space offer an excellent framework for understanding how typical confounders, arising from both subject variability and acquisition noise, are integrated into the model. This significantly contributes to a more explainable and robust model, enabling the disentanglement and characterization of common confounding factors —such as brain size or orientation—within the latent representation, treating them as informative components rather than as noise Alfaro-Almagro et al. (2021). As illustrated in Fig. 10, affine transformations are encoded within almost every latent variable, accounting for translations (10a), rotations (10b,10c), and scaling (10d) due to varying brain shapes and positions during acquisition. Moreover, we also found that intensity variations are encoded within these variables. We observe that some variables (e.g., latent variable 3) display a skull-stripping effect. On the other hand, we also observe a general decrease in intensity for specific brain areas. This variability could be attributable to factors such as differences in acquisition timing (e.g., delayed imaging leading to lower uptake) or variations in administered dose, which can differ between subjects in clinical PET protocols Murthy et al. (2020); Doot et al. (2010). Although intensity normalization is applied to mitigate such inter-subject variability, residual effects might still persist, potentially impacting the observed signal in specific regions.

Finally, we also performed a logistic regression trained on the learned representations, achieving excellent values of the metrics, although classification is not the main objective of this work. We found that the performance, shown in Table 1 and Fig. 8 is comparable to the previous studies Wakefield et al. (2024); Bit et al. (2024); Vivek et al. (2023). Furthermore, the dementia-related variable explains the majority of the classification’s predictive power, whereas the remaining latent variables perform poorly, indicating that the VAE model primarily encodes dementia-related information withing the similarity-related variable.

Overall, this work presents a flexible and generalizable semi-supervised VAE framework that effectively integrates clinical variables through a similarity-driven regularization. By incorporating noise as part of the latent representation, the model not only captures meaningful disease-related patterns but also accounts for subject variability in an interpretable way. We believe this approach provides a valuable tool for advancing the study of dementia with neuroimaging data.

## Figures and Tables

**Figure 1:**
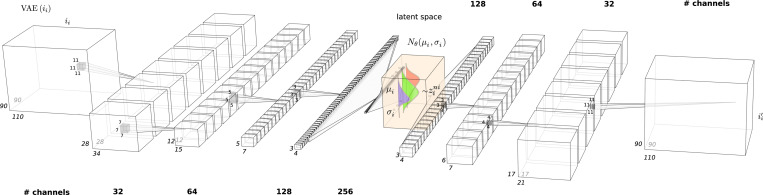
Architecture of the VAE model. Convolutional layers extract feature from high dimensional volumes into feature maps, where are hierarchically compressed into lower representations.

**Figure 4:**
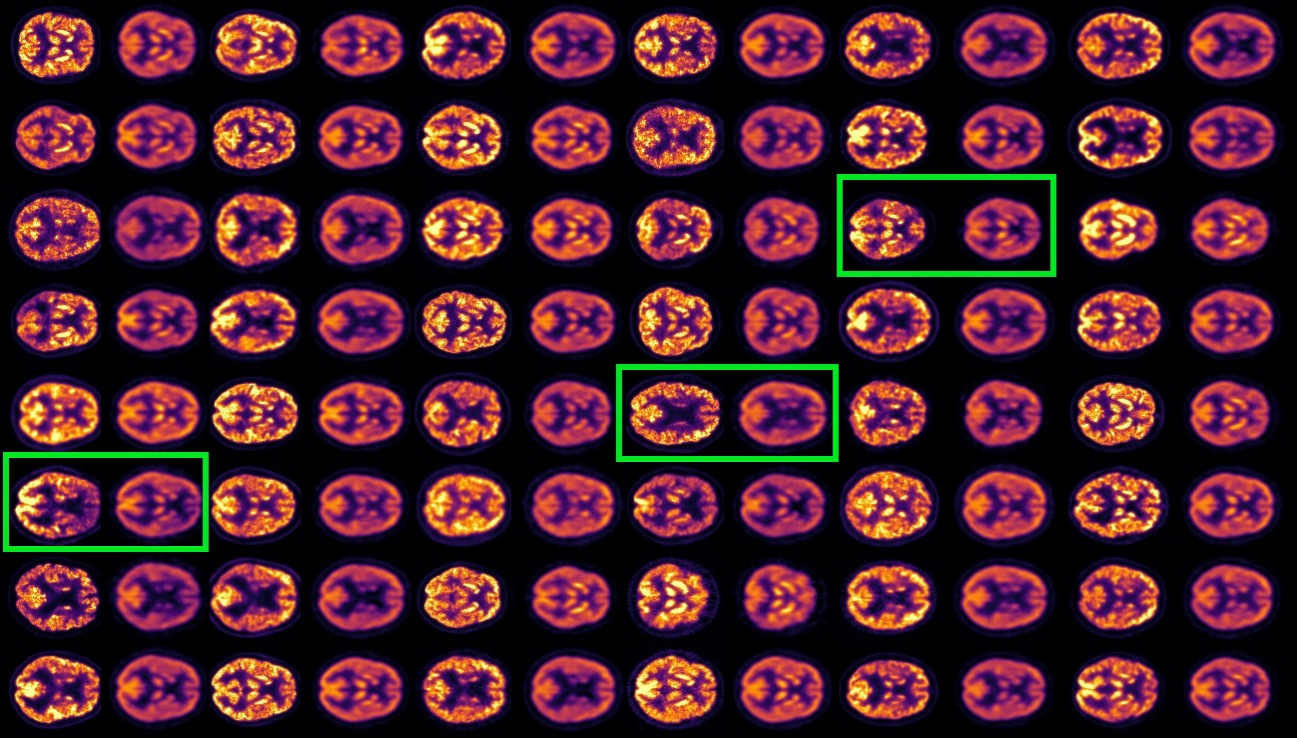
Odd columns: input brain slices. Even columns: VAE reconstructions. Green boxes highlight input-reconstruction pairs. Color represents intensity; reconstructions show lower intensity but preserve relevant structure. The KL regularization counterweights the reconstruction, producing lower quality scans than the input.

**Table 1:** Classification metrics for the AD vs. HC task. The values of our model were validated using bootstrap validation with 10 resamples. We also provide a comparison with work from the literature.

Metric	Our model	ADAS13	Wakefield et al. (2024)	Dolci et al. (2024)

Accuracy	0.8 ± 0.02	0.96 ± 0.03	-	0.926 ± 0.02
Sensitivity (recall)	0.79 ± 0.04	0.95 ± 0.01	-	0.876 ± 0.03
Specificity	0.77 ± 0.02	0.95 ± 0.02	-	-
Balanced Accuracy	0.79 ± 0.02	0.96 ± 0.02	0.85 ± 0.01	-
